# A negative feedback loop involving NF-κB/TIR8 regulates IL-1β-induced epithelial- myofibroblast transdifferentiation in human tubular cells

**DOI:** 10.1007/s12079-021-00620-8

**Published:** 2021-05-04

**Authors:** Keguo Jiang, Yuying Zhang, Fan He, Mingming Zhang, Tianyu Li, Zhenzhen Tu, Deping Xu, Min Zhang, Linzi Han, Liyu Guo, Haisheng Zhou, Deguang Wang

**Affiliations:** 1 Department of Nephrology, The Second Affiliated Hospital, Anhui Medical University (AHMU), No. 678 Fu Rong Road, Hefei, China; 2 Department of Biochemistry and Molecular Biology, AHMU, No. 69 Mei Shan Road, Hefei, China; 3 Clinical Laboratory, The Affiliated Hefei Hospital, AHMU, No. 246 Hepin Rd., Hefei, China; 4 Clinical Laboratory, The First Affiliated Hospital, AHMU, No. 81 Meishan Rd., Hefei, China; 5 Center for Scientific Research, AHMU, No. 69 Mei Shan Road, Hefei, China; 6 Department of Nephrology, The Third Affiliated Hospital of Anhui Medical University, No. 390, Huai He Road, Hefei, China

**Keywords:** Epithelial‐myofibroblast transdifferentiation, Interleukin-1β, TIR8, NF-κB

## Abstract

Renal tubular epithelial-myofibroblast transdifferentiation (EMT) plays a central role in the development of renal interstitial fibrosis (RIF). The profibrotic cytokine interleukin (IL)-1 and the IL-1 receptor (IL-1R) also participate in RIF development, and Toll/IL-1R 8 (TIR8), a member of the Toll-like receptor superfamily, has been identified as a negative regulator of IL-1R signaling. However, the functions of TIR8 in IL-1-induced RIF remain unknown. Here, human embryonic kidney epithelial cells (HKC) and unilateral ureteric obstruction (UUO)-induced RIF models on SD rats were used to investigate the functions of TIR8 involving IL-1β-induced EMT. We showed that IL-1β primarily triggers *TIR8* expression by activating nuclear factor-κB (NF-κB) in HKC cells. Conversely, high levels of TIR8 in HKC cells repress IL-1β-induced NF-κB activation and inhibit IL-1β-induced EMT. Moreover, in vitro and in vivo findings revealed that TIR8 downregulation facilitated IL-1β-induced NF-κB activation and contributed to TGF-β1-mediated EMT in renal tubular epithelial cells. These results suggested that TIR8 exerts a protective role in IL-1β-mediated EMT and potentially represents a new target for RIF treatment.

**Supplementary Information:**

The online version contains supplementary material available at 10.1007/s12079-021-00620-8.

## Introduction

In all forms of progressive kidney disease, the development of renal interstitial fibrosis (RIF) characterized by tubular injury and interstitial fibrosis most reliably predicts the progression toward end-stage renal failure (Meyer 2003); however, little is known about the precise underlying mechanisms. Renal tubular epithelial–myofibroblast transdifferentiation (EMT) is thought to play a central role in RIF development (Acloque et al. 2009; Jinde et al. 2001).

Interleukin (IL)-1 is a proinflammatory, profibrotic cytokine that exerts a wide range of biological effects, including stimulation of proliferation, production of extracellular matrix, induction of leukocyte chemotactic molecule release, augmentation of adhesion molecule expression, and induced release of nitric oxide, reactive oxygen species, and transforming growth factor (TGF)-β (Vesey et al. 2002). Additionally, IL-1 is implicated in animal models or human forms of chronic kidney disease, with *IL-1* expression significantly elevated in experimental glomerulonephritis, whereas blocking IL-1 activity suppresses acute glomerular injury and inhibits RIF (Chen et al. 1997). Moreover, IL-1 is capable of inducing morphological and phenotypic transdifferentiation of a normal rat tubular epithelial cell line into myofibroblast-like cells (Vesey et al. 2002). IL-1β, as a much stronger inducer of nuclear factor (NF)-κB response, interacts with IL-1 receptor (IL-1R) to induce EMT by activating the IL-1/IL-1R/NF-κB signaling pathway (Dinarello and Wolff 1993; Gasse et al. 2007; Zhang et al. 2015). The NF-κB complex is usually inactive and located in the cytoplasm while bound to IκB inhibitor proteins; however, after activation of IL-1/IL-1R/NF-κB signaling, NF-κB is activated and translocates to the nucleus, where it forms the p65/p50 complex to control target gene transcription (Gupta et al. 2010; Zheng et al. 2011). Recently, a positive correlation between NF-κB activation and EMT induction was described in several human cancers (Pires et al. 2017); however, there is no evidence of NF-κB-regulated EMT induction associated with RIF development.

Toll/IL-1R 8 (TIR8), also known as single immunoglobulin IL-1R-associated protein (SIGIRR), is ubiquitously expressed in tissues, particularly in the kidney, digestive tract, liver, lung, and lymphoid organs. In the kidney, it is expressed on the luminal border and basolateral membrane of the proximal tubular cells (Riva et al. 2012). However, the stimuli that induce *TIR8* expression are not yet well defined, and the involved mechanisms are unknown. TIR8 is a member of the IL-1R superfamily (Garlanda et al. 2004) and a fringe member of a family with structural features incompatible with conventional signaling. Additionally, TIR8 has been identified as a negative regulator of the IL-1R signaling pathway (Garlanda et al. 2007; Nam 2006); however, a correlation between activated IL-1R signaling and *TIR8* expression remains poorly defined, especially with respect to a TIR8-related regulatory mechanism involved in IL-1β-induced EMT in RIF development.

Therefore, we hypothesized that *TIR8* expression is regulated by NF-κB and potentially involved in inhibiting NF-κB-dependent EMT induction in human renal tubular epithelial (HKC) cells. We tested this hypothesis by activating IL-1β/IL-1R/NF-κB signaling *in vitro* and demonstrating a negative feedback loop involving NF-κB/TIR8, which regulated in vivo IL-1β-induced EMT of tubular cells. The results showed that TIR8, as a negative regulator of the IL-1R signaling, might play a protective role in RIF development and potentially represent a promising new target for RIF treatment.

## Materials and methods

### Reagents, plasmids, and antibodies

Recombinant human IL-1β was purchased from Sigma-Aldrich (#SRP3083, St. Louis, MO, USA). The GV248 lentiviral short-hairpin (sh) RNA-expression vector targeting human NF-κB (p65; shp65), human TIR8 (shTIR8), scrambled shRNA (shSc), and the GV248 lentiviral vector were obtained from Genechem (Shanghai, China). The target sequences are listed in Supplementary Table 1. Packaging vectors (pVSVG, pREV, and pMDL) were used as described previously (Zha et al. 2015). Human *TIR8* cDNA was subcloned at *Bam*HI and *Eco*RI sites of the pcDNA3.1-C/DYK expression vector.

Primary antibodies used for western blotting analysis include, TIR8 (Abcam, #ab228977. Cambridge, MA, USA), E-cadherin (CST, #14,472. Shanghai, China), vimentin (CST, #5741), p65 (CST, #8242s), phosphorylated p65 (p-p65 at Ser536. CST, #3031s), TGF-β1 (Proteintech, #21898-1-AP. Rocky Hill, NJ, USA), glyceraldehyde 3-phosphate dehydrogenase (GAPDH, Image Bioscience, #IMA1004L. Beijing, China). Primary antibodies against E-cadherin (CST, #14,472), vimentin (CST, #5741), p-p65 (Santa Cruz, #SC136548. CA, USA), TIR8 (Abcam, #ab228977), IL-1β (Wanleibio, #WL00891. Shenyang, China), and TGF-β1 (Wanleibio, #WL02193) were used to stain the corresponding proteins, and IgG (CST, #4414S) was used as a negative control.

### Cell lines, cell culture, lentivirus production, and transduction

HKC epithelial cells were obtained from Peking Union Medical College (Beijing, China). HEK 293T packaging cells have been described previously (Zha et al. 2015). The HKC cells were grown in Dulbecco’s modified Eagle medium (DMEM)/F12 supplemented with 10% fetal bovine serum (FBS). HEK 293T cells were maintained in DMEM supplemented with 10% FBS and transfected with either of the recombinant vectors or the corresponding control vectors together with packaging plasmids (pVSVG, pREV, and pMDL). Culture supernatants with lentivirus were collected at 48-h post-transfection and used to infect target cells, as described previously (Zha et al. 2015). The recombinant vectors with the human *TIR8* cDNA were transiently transfected into HKC cells using Lipofectamine 3000 (Invitrogen, Carlsbad, CA, USA) according to manufacturer instructions. All of the cells were cultured at 37 °C in a humidified incubator with 5% CO_2_.

### Semi‐quantitative reverse transcription polymerase chain reaction (qRT-PCR)

Total RNA isolation, reverse transcription, and semi-quantitative PCR analysis to determine mRNA expression was performed as described previously (Zha et al. 2015). The primer sequences are listed in Supplementary Table 2.

### Western blot and immunofluorescence analyses

Western blot analysis to determine protein levels was performed as described previously (Zha et al. 2015). Semi-quantitative data from densitometric analysis of proteins were obtained using the Gel-Pro analyzer software (https://omictools.com/gel-pro-analyzer-tool). Indirect immunofluorescence staining was performed using an established procedure on cells cultured in 24-well plates. Briefly, cells were treated accordingly, fixed with 4% (w/v) paraformaldehyde for 15 min, blocked with phosphate-buffered saline (PBS) containing 5% goat serum and 0.1% Triton X-100 for 20 min at room temperature, and incubated with specific primary antibodies (CST China). The cells were then routinely stained with fluorescein isothiocyanate-conjugated secondary antibodies (CST China). Cells were also stained with 4′,6-diamidino-2-phenylindole for 3 min to visualize nuclei. Fluorescence was observed and photographed using an Axio Observer 3 inverted fluorescence microscope (Carl Zeiss, Oberkochen, Germany).

### Scratch‐wound and transwell assays

Cell motility and migration were evaluated using scratch-wound and Transwell assays, respectively. HKC/shSc cells and HKC/shTIR8 cells were cultured until near confluency in 6-well plates in the presence of IL-1β (10 ng/ml), and monolayers were wounded by scratching with a 2-µl pipet tip. Cells were then incubated in the fresh medium with 2% FBS and 10 ng/ml IL-1β for up to 48 h after wounding. Images of the same area were obtained using a Celldiscoverer 7 (Carl Zeiss, Oberkochen, Germany) at four separate time points (0, 12, 24, and 48 h) along each scratch. Image Pro Plus software (v.6.2; Media Cybernetics, Silver Spring, MD, USA) was used to determine the size of the wound in each image, as well as compare images at each time point with those acquired initially. All experiments were performed in triplicate.

Cells (5 × 10^4^) were seeded onto the filters of tissue-culture-treated Transwell plates (8-µm pore size, 0.33-cm^2^ growth area; Corning, Corning, NY, USA). After 48 h of incubation with IL-1β (10 ng/ml) at 37 °C, filters were fixed with 3% paraformaldehyde in PBS and stained with 0.1% Coomassie Blue in 10% methanol and 10% acetic acid, and the upper surface of the filter was carefully wiped with a cotton-tipped applicator. Cells that passed through the pores were counted and photographed in five fields. Culture experiments were performed in triplicate.

### Experimental models

SD rats were obtained from the Experimental Animal Center of Anhui Medical University of China. The study were performed in compliance with the Guide for the Care and Use of Laboratory Animals and the ethical guidelines of Anhui Medical University. Experimental UUO was performed on SD rats to establish experimental models of UUO-induced renal fibrosis. Anesthesia was induced with 2% Nembutal (3.0 ml/kg), during which the left ureter of each animal was ligated with 4−0 silk at the ureteropelvic junction through a right-flank incision, followed by wound closure in layers. Rates in the sham-operated group had their ureters exposed and manipulated without ligation. After operation, rats were allowed to recover with free access to food and water. Kidneys were harvested at day 21, with 24-h urine and blood samples collected to detect kidney function before termination. The animals were randomly divided into three groups, including control (*n* = 2), sham-operated (*n* = 4), and UUO (*n* = 11) groups.

### Immunohistochemistry, hematoxylin & eosin (H&E) staining, and masson trichrome staining

Sections from paraffin-embedded tissues were prepared at 4-µm thickness and incubated with anti-IL-1β (1:100), anti-TGF-β1 (1:100), anti-TIR8 (1:500), anit-p-p65 (1:50), anti-E-cadherin (1:100), or anti-vimentin (1:200) antibodies overnight at 4 °C, followed by incubation with the appropriate secondary antibody for 1 h at room temperature. Sections were then stained with H&E to assess renal injury, and kidney fibrosis was assessed on kidney sections stained with Masson trichrome staining. Blue staining areas represented collagen deposition.

### Statistical analysis

Data were analyzed by one-way analysis of variance using GraphPad Prism software (v.5.0; GraphPad Software, La Jolla, CA, USA). Results are expressed as the mean ± standard error of the mean, and a *P* < 0.05 was considered statistically significant.

## Results

### IL-1β regulates TIR8 expression in HKC cells in connection with NF-κB activation

**Fig. 1:**
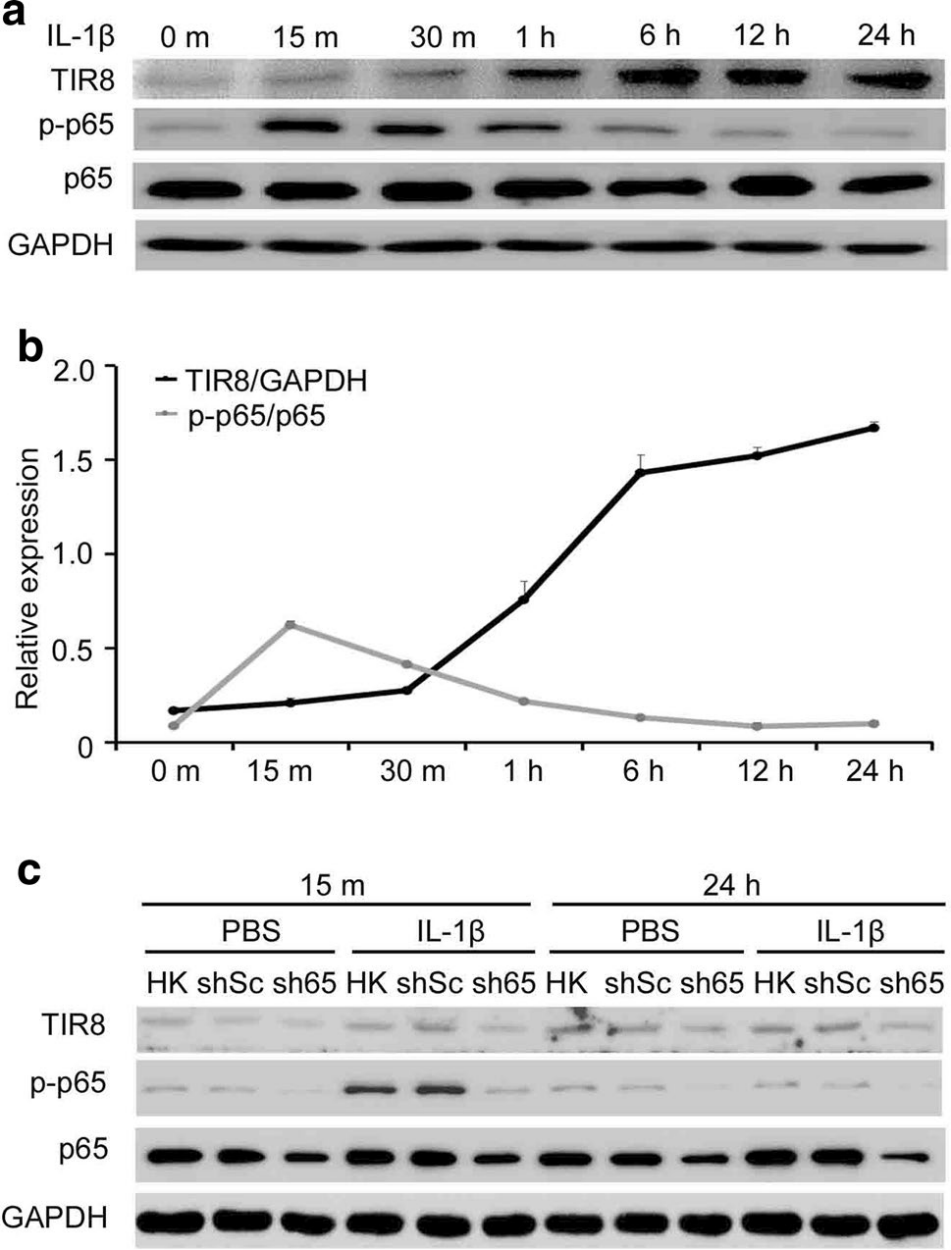
IL-1β induces *TIR8* expression in HKC cells in connection with NF-κB activation. **a** Western blot analysis of TIR8 levels and NF-κB activation (p-p65 levels) in HKC cells treated with 10 ng/ml IL-1β for 0 m, 15 m, 30 m, 1 h, 6 h, 12 h, and 24 h. **b** Semi-quantitative data from densitometric analysis of TIR8 and p-p65 levels are presented as the relative ratio of each protein to GAPDH and p65, respectively. **c** Western blot analysis of IL-1β-induced TIR8 levels and NF-κB activation in HKC cells transfected with shp65 or shSc

To investigate whether IL-1β regulates *TIR8* expression in HKC cells, immunoblot analysis was performed to detect TIR8 protein levels and NF-κB activation in HKC cells treated with IL-1β (10 ng/ml) for 15 m, 30 m, 1 h, 6 h, 12 h, and 24 h. The results showed that *TIR8* expression was significantly and continuously increased in HKC cells by exposure to IL-1β, whereas p-p65 levels increased during 15 m of IL-1β induction and then decreased gradually after exposure to IL-1β for 30 m to 24 h (Fig. 1a and b). To determine whether activated NF-κB directly regulates *TIR8* expression, HKC cells were transfected with shp65 and then exposed to IL-1β for 15 m and 24 h. Endogenous *TIR8* expression was diminished following p65 knockdown (Fig. 1c).

### Ectopic TIR8 expression negatively regulates IL-1β-induced NF-κB activation in HKC cells

**Fig. 2:**
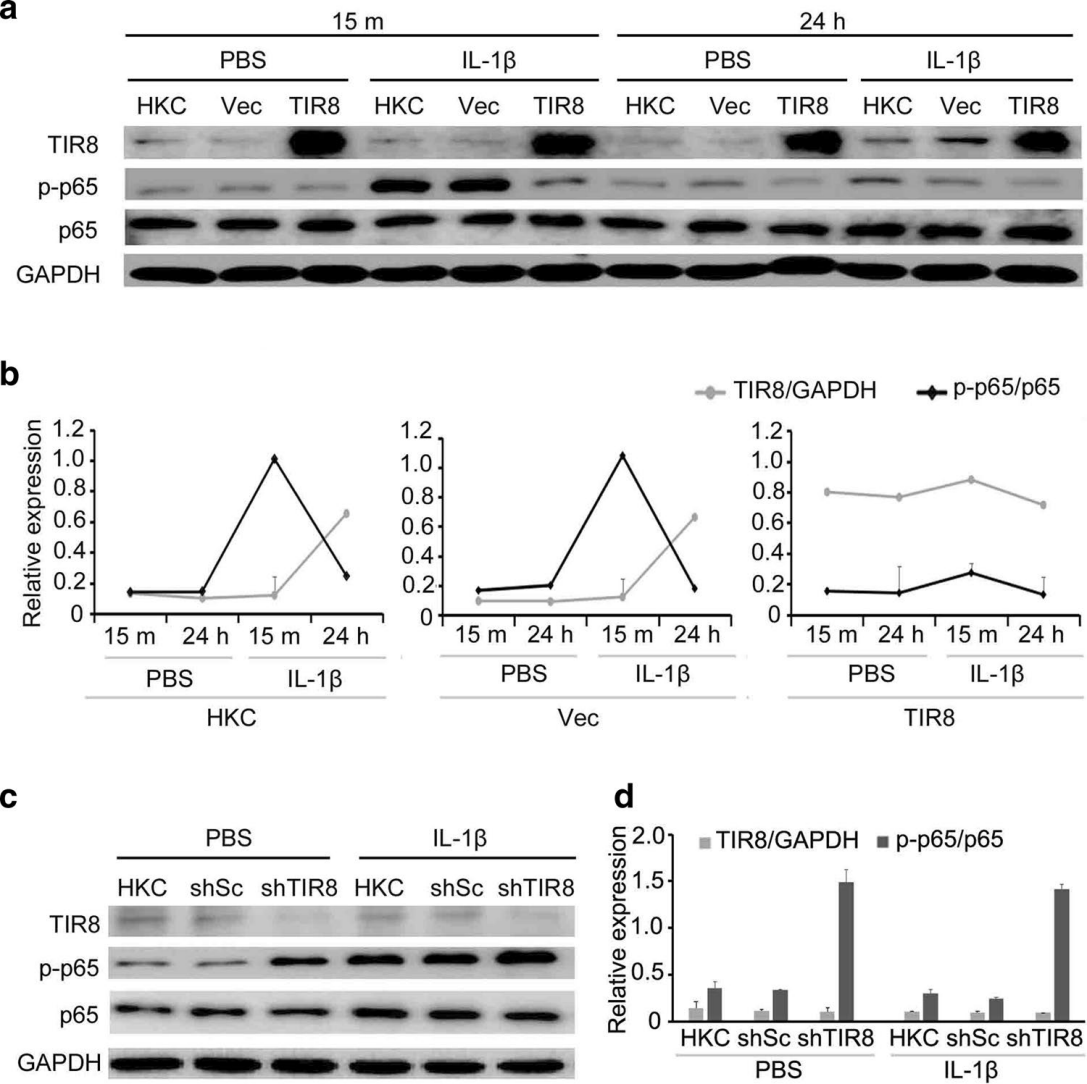
Ectopic expression of TIR8 negatively regulates IL-1β-induced NF-κB activation in HKC cells. **a, b** Immunoblot analysis of TIR8 levels and NF-κB activation (p-p65 levels) in HKC cells overexpressing *TIR8* in response to IL-1β. **c, d** Western blot analysis of TIR8 levels and NF-κB activation in HKC/shTIR8 cells following exposure to IL-1β for 15 m

TIR8 is a negative regulator of the IL-1R signaling pathway (Garlanda et al. 2007; Nam 2006; Polentarutti et al. 2003); therefore, we hypothesized that *TIR8* overexpression might negatively regulate IL-1β/IL-1R signaling. To test this hypothesis, we established an HKC cell line overexpressing *TIR8* (HKC/*TIR8*). Immunoblot analysis revealed no alterations on p-p65 levels in HKC/*TIR8* cells after exposure to IL-1β (Fig. 2a and b). We then established a stable *TIR8*-knockdown HKC cell line (HKC/shTIR8) to attenuate TIR8 level/activity in HKC cells. Interestingly, western blot analysis showed dramatic increases in p-p65 levels in HKC/shTIR8 cells both in the presence and absence of IL-1β within 15 m (Fig. 2c and d), indicating that ectopic *TIR8* expression negatively regulated IL-1β-induced NF-κB activation in HKC cells.

### TIR8 plays a protective role in IL-β-induced EMT in HKC cells

**Fig. 3:**
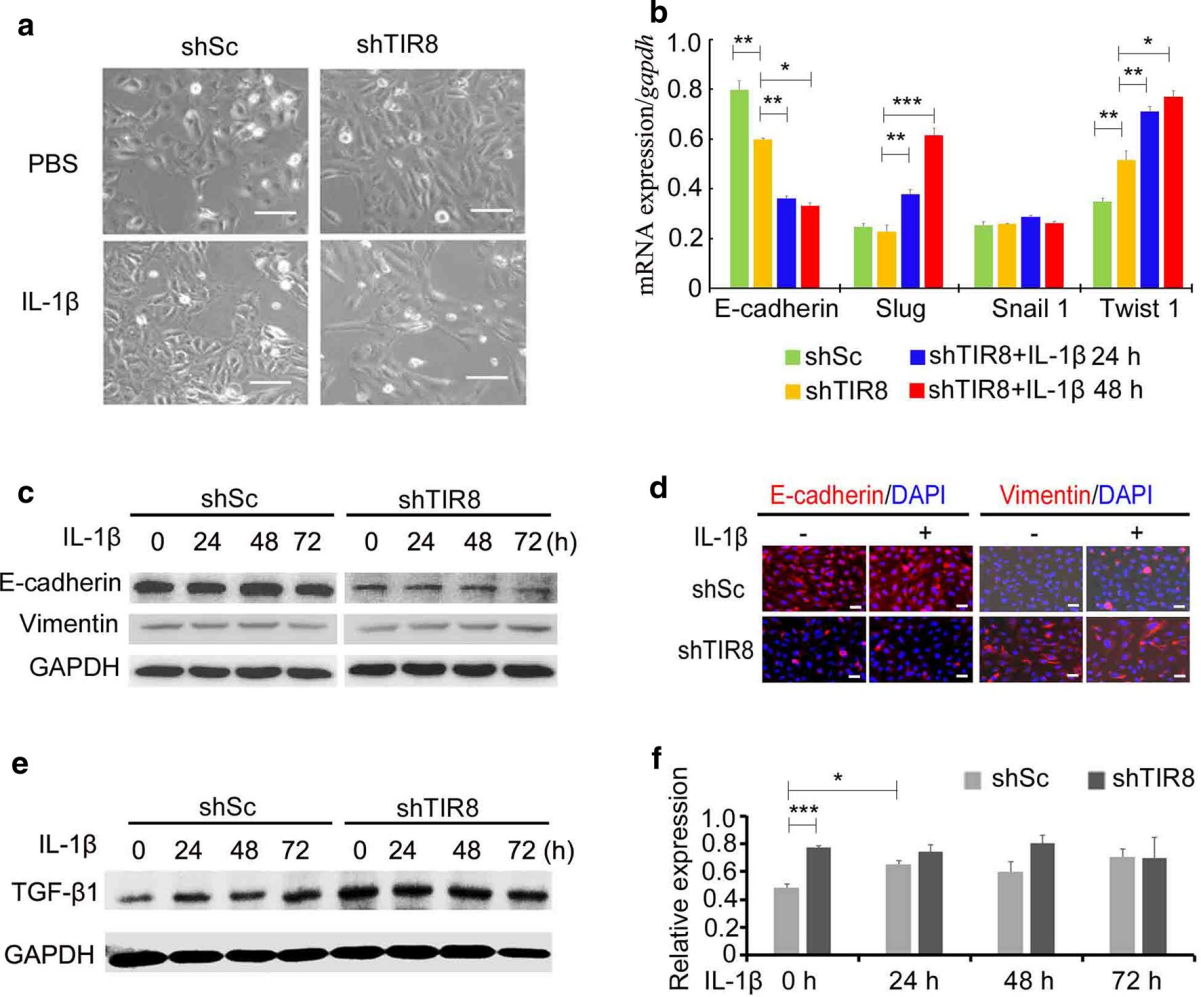
Attenuated *TIR8* expression facilitates IL-1β-induced EMT in renal tubular epithelial cells. **a** Morphological alteration of HKC/shTIR8 cells treated with IL-1β (10 ng/ml) for 72 h (scale bars: 20 μm). **b** qRT-PCR analysis of gene expression in HKC/shTIR8 cells treated with IL-1β (10 ng/ml) for 24 and 48 h. *P < 0.05, **P < 0.01, ***P < 0.001. **c** Immunoblot analysis of E-cadherin and vimentin levels in HKC/shTIR8 cells treated with IL-1β for 24 h, 48 h, and 72 h. **d** Immunofluorescence analysis of E-cadherin and vimentin levels in HKC/shTIR8 cells treated with IL-1β for 72 h (scale bars: 20 μm). **e, f** Immunoblot analysis of TGF-β1 levels in HKC/shTIR8 cells treated with IL-1β for 24 h, 48 h, and 72 h. *P < 0.05, ***P < 0.001

A previous study suggested that IL-1 induces transdifferentiation of tubular epithelial cells in renal fibrosis (Torbohm et al. 1989). To evaluate the activation of IL-1β/IL-1R/NF-κB signaling in HKC cells, we evaluated IL-1β-induced EMT. First, to observe alterations in cell morphology, HKC/shSc and HKC/shTIR8 cells were cultured in serum-free medium for 3 days with or without IL-lβ, respectively. As expected, *TIR8*-knockdown both in the presence and absence of IL-lβ displayed significant hypertrophy and lost the cobblestone morphology (Fig. 3a); however, there was less morphological alteration observed in HKC/shSc cells in the presence of IL-lβ.

EMT is associated with loss of the epithelial marker E-cadherin and gain of the mesenchymal markers vimentin, SNAI1 (Snail), Snail homolog protein 2 (Slug), and Twist-related protein 1 (TWIST1). qRT-PCR to detect mRNA levels of these genes in both HKC/shSc and HKC/shTIR8 cells following IL-1β induction revealed decreases in *E-cadherin* expression at 24 and 48 h (Fig. 3b), whereas IL-1β induced *Slug* and *Twist1* expression in HKC/shTIR8 cells, although no significant change was noted in the expression of these genes in HKC/shSc cells (data not shown). Both immunoblot analysis and immunofluorescence assay showed that IL-1β attenuated E-cadherin levels and upregulated vimentin levels in HKC cells (Fig. 3c and d).

Because TGF-β1 is used as a canonical inducer to promote TWIST1 and Slug levels required for EMT, we detected changes in TGF-β1 levels in HKC cells by immunoblot analysis. In agreement with a previously report that IL-1β increases expression of TGF-β1 (Hao et al. 2011), we observed significant increases in TGF-β1 levels in HKC cells treated with IL-1β for 24 and 72 h. Furthermore, we detected higher levels of TGF-β1 in HKC/shTIR8 cells in both the presence and absence of IL-1β (Fig. 3e).

**Fig. 4:**
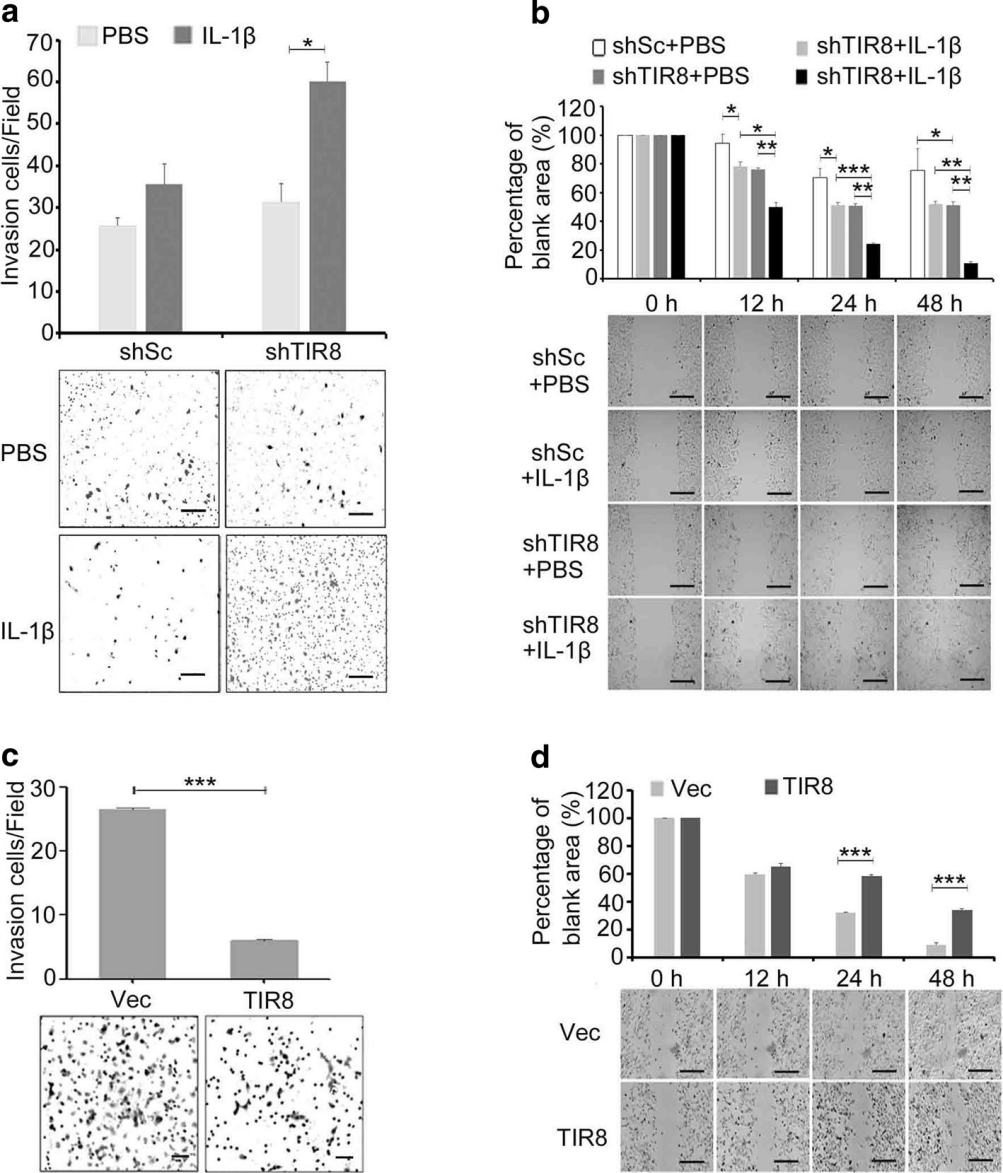
Ectopic expression of *TIR8* regulates motility and invasion of HKC cells.** a** Transwell assay evaluating the invasive ability of HKC cells overexpressing *TIR8* (scale bars: 10 μm). ***P < 0.001. **b** Analysis of the migration of HKC/shTIR8 cells treated with IL-1β for 12 h, 24 h, and 48 h (scale bars: 10 μm). *P < 0.05, **P < 0.01, ***P < 0.001. **c** Analysis of the invasive ability of HKC/shTIR8 cells treated with IL-1β for 48 h (scale bars: 10 μm). *P < 0.05. **d** Scratch-wound assays evaluating the migration of HKC cells overexpressing *TIR8* (scale bars: 10 μm). ***P < 0.001

EMT is associated with altered cell migration and invasion; therefore, we performed *in vitro* Transwell migration assays to investigate the invasive capacity of IL-1β-induced HKC cells. The results indicated that HKC/shTIR8 cells showed a significant increase in invasive capacity as compared with that of HKC/shSc cells following exposure to IL-1β for 48 h (Fig. 4a). Additionally, a wound healing assay to assess HKC cell motility revealed increased an increased number of HKC/shTIR8 cells moving into the scratch wound in the presence of IL-1β over 12 h, 24 h, and 48 h relative to that observed by HKC/shSc cells (Fig. 4b). However, *TIR8* overexpression inhibited cell migration and invasion in the absence of IL-1β (Fig. 4c and d).

### **IL-1β activates NF-κB and promotes TGF-β1-mediated EMT associated with TIR8 downregulation in an unilateral ureteric obstruction (UUO)-induced renal fibrosis model**

**Fig. 5:**
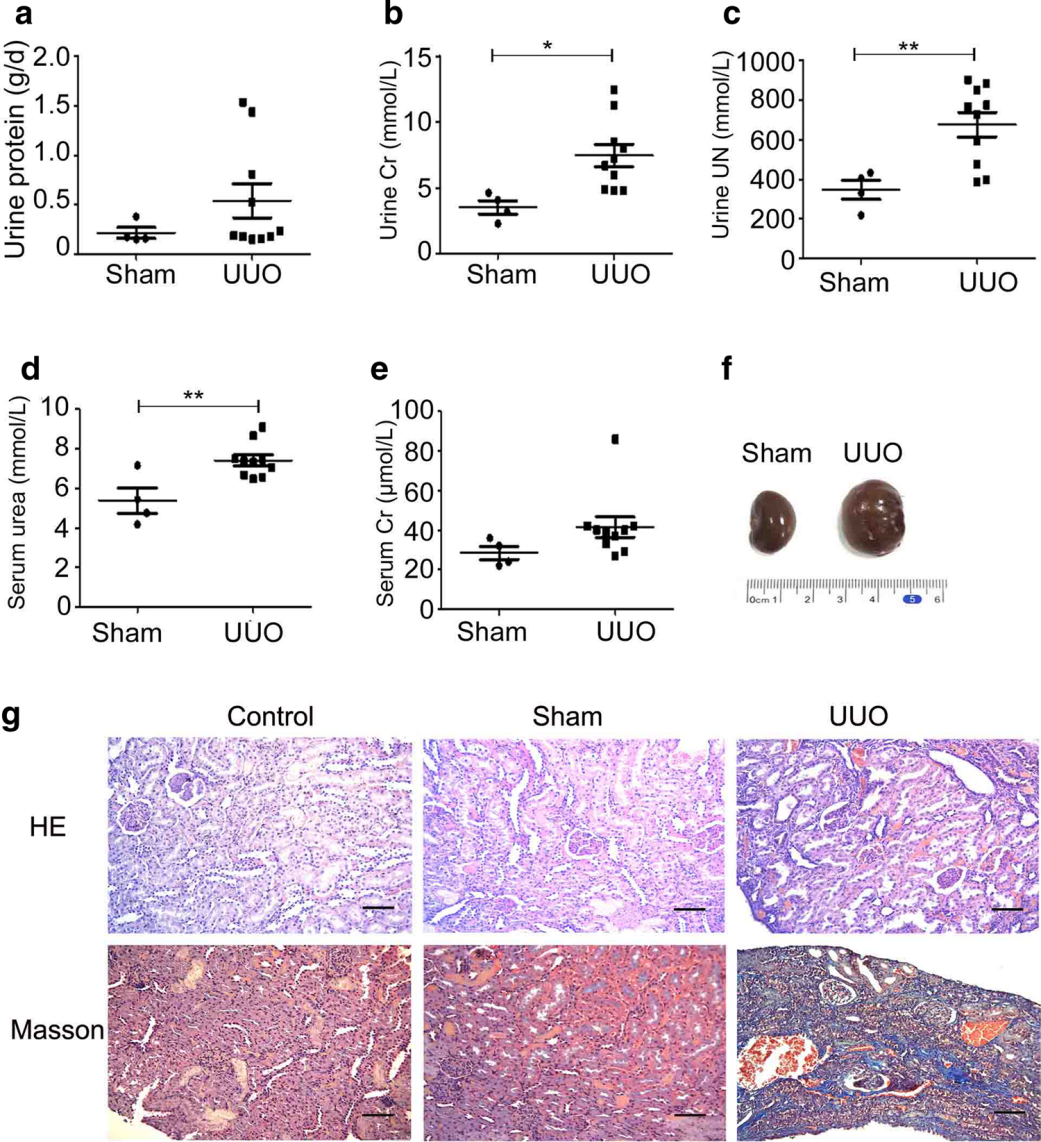
Establishment of UUO-induced renal fibrosis animal models.** a-e** Analysis of kidney function in the sham-operated (*n* = 4) and UUO (*n* = 11) groups. *P < 0.05, **P < 0.01 versus the sham-operated group. Cr, creatinine, UN, urea nitrogen. **f** Representative morphology of sham-operated (Sham) and UUO kidneys. G. H&E staining to evaluate renal histopathology and Masson trichrome staining to assess tubulointerstitial fibrosis (scale bars: 20 μm)

We established UUO-induced renal fibrosis animal models on SD rats to identify whether a negative feedback loop involving NF-κB/TIR8 regulates in vivo IL-1β-induced EMT of tubular cells. Analysis of kidney function showed no dramatic alterations in 24-h urinary protein or serum urea between the sham-operated and UUO groups, whereas levels of serum urea and 24-h urinary creatinine, as well as 24-h urinary urea nitrogen, were significantly increased in the UUO group relative to the sham-operated group (Fig. 5a–e and Table 1). Additionally, kidneys in the UUO group were characterized by significant renal swelling, thinning of renal parenchyma, and hydronephrosis (Fig. 5f), whereas H&E staining showed normal morphological structures in renal tissue sections from both the control and the sham-operated groups. Moreover, kidney damage in the forms of tubular dilatation, epithelial cell necrosis, hemorrhage, and inflammatory cell infiltration were observed in renal tissues from the UUO group (Fig. 5g), and collagen deposition was significantly increased in obstructed kidneys based on Masson trichrome staining. These findings confirmed establishment of the UUO-induced renal fibrosis model based on glomerular and tubular functions.

**Table 1:** Analysis of kidney function in the sham-operated and UUO rats

Group	Urine protein (g/d)	Urine Cr (mmol/L)	Urine urea nitrogen (mmol/L)	Serum urea (mmol/L)	Serum Cr (µmol/L)
UUO (*n* = 4)	0.54 ± 0.54	7.4 ± 2.7	677.01 ± 198.54	7.42 ± 0.86	41.50 ± 16.5
Sham (*n* = 11)	0.22 ± 0.11	3.5 ± 1.0	346.875 ± 96.59	5.39 ± 1.30	28.50 ± 6.61
*P* value	0.099	0.016	0.009	0.005	0.057

**Fig. 6:**
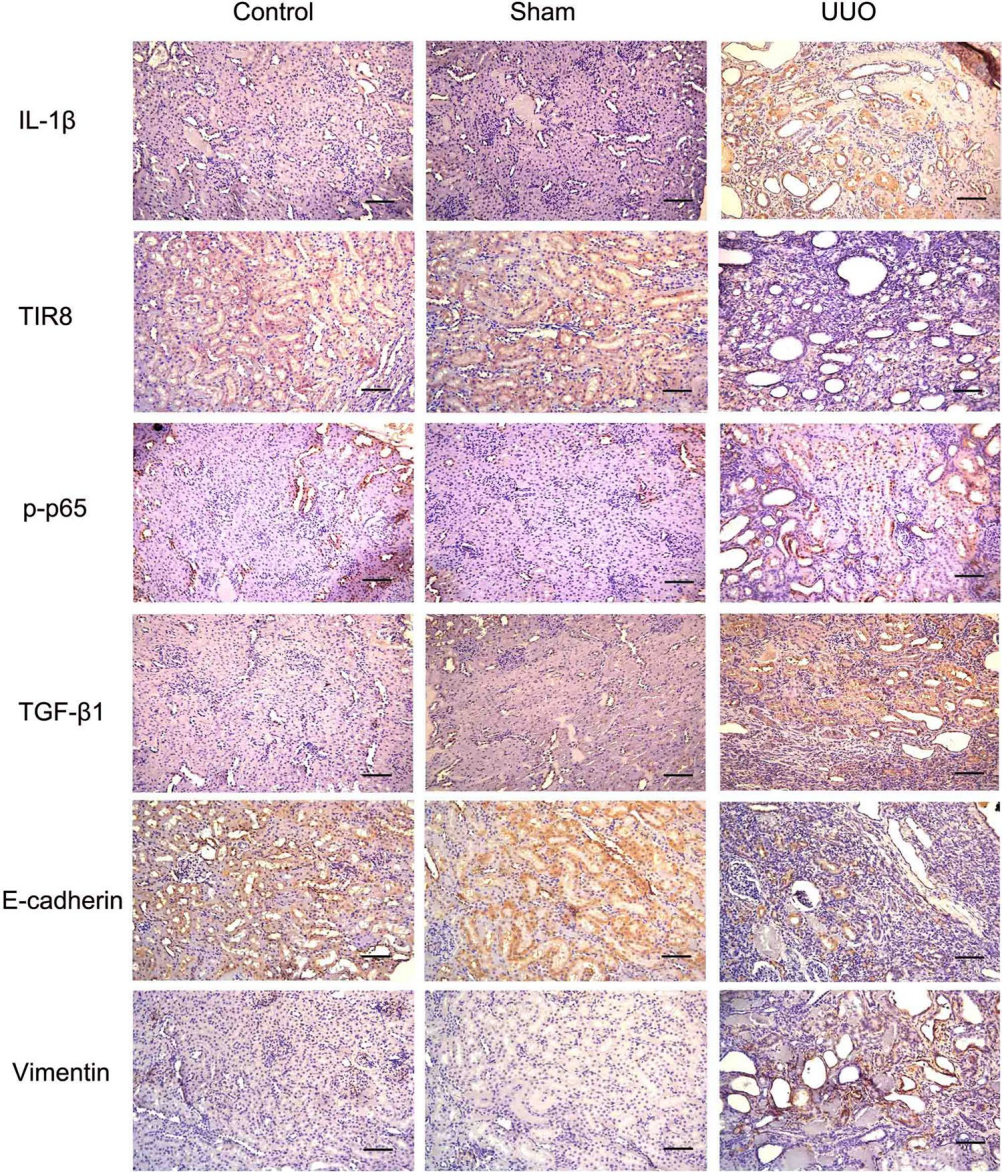
IL-1β activates NF-κB signaling and promotes TGF-β1-mediated EMT in connection with TIR8 downregulation in UUO-induced renal fibrosis. Immunohistochemical analysis of IL-1β, TIR8, p-p65, TGF-β1, E-cadherin, and vimentin levels in renal tissues (scale bars: 20 μm). Control, normal kidney; Sham, sham-operated group; UUO, UUO-treated group

Immunohistochemistry analysis to detect levels of IL-1β, TIR8, and activated-NF-κB (p-p65) revealed high levels of both IL-1β and p-p65 in renal tissues from the UUO group, whereas weak cytoplasmic IL-1β and nuclear p-p65 immunoreactivity was detected in renal tissues from both control and the sham-operated groups (Fig. 6). According to our *in vitro* findings, upregulated TGF-β1 levels induced by IL-1β in kidneys following UUO might play central roles in initiation and progression of renal fibrosis. As expected, we observed elevated TGF-β1 levels in tubular epithelial cells from UUO kidney tissue sections. Coincident with upregulated TGF-β1, immunohistochemistry analysis showed decreases in E-cadherin levels, as well as increases in vimentin levels. Importantly, we confirmed attenuated levels of TIR8 in renal tubular epithelial cells from the UUO group.

## Discussion

IL-1β interacts with IL-1R and activates NF-κB to promote a wide range of biological effects capable of inducing morphological and phenotypic transdifferentiation of tubular epithelial cells into myofibroblast-like cells (Gasse et al. 2007; Stewart and Marsden 1995; Vesey et al. 2002). TIR8, a member of the IL-1R superfamily, is a potentially important regulator involved in this process. In the present study, we observed increased *TIR8* expression in HKC cells treated with IL-1β over a 24-h period along with increases in p-p65 levels after 15 m of IL-1β induction, followed by a gradual decrease from 30 m to 24 h. *TIR8* knockdown to investigate TIR8-related physiological functions associated with IL-1/IL-1R signaling revealed that IL-1β continually activated IL-1R/NF-κB-related signaling in HKC/shTIR8 cells but not in normal HKC cells. To evaluate regulation of *TIR8* expression by p-p65 (activated NF-κB) following activation of IL-1R/NF-κB signaling, we knocked down p65 and found that activated NF-κB promoted *TIR8* expression in HKC cells in the presence of IL-1β.

Previous studies reported several molecules that negatively regulate IL-1R-mediated signaling to maintain a balance between inhibition and activation of the immune system (Divanovic et al. 2005; Thomassen et al. 1999). Some of these molecules are present constitutively to control IL-1R activation at the physiological level, whereas others are upregulated by IL-1R signaling in the presence of inflammatory stimuli to weaken the IL-1R response as part of a negative feedback loop (Li and Qin 2005; Qin et al. 2005). In this regard, IL-1R activation is a double-edged sword, with negative regulation of IL-1R signaling potentially required to avoid a continuous inflammatory response. This suggests a fine balance between regulation of short- and long-term inflammatory signaling in HKC cells, with this activity possibly involving TIR8. Our findings demonstrated that constitutive expression of TIR8 on the surface of HKC cells appeared to maintain a balance in immune-related signaling. Conversely, *TIR8* overexpression induced by activated NF-κB showed no significant effects on the activation of IL-1R/NF-κB signaling induced by IL-1β in HKC cells.

Previous studies indicate that glomerular and tubulointerstitial expression of IL-1 is elevated in glomerulonephritis (Niemir et al. 1997; Nikolic-Paterson et al. 1996). Additionally, Lemos et al. (2018) reported that IL-1 upregulation in human glomerulonephritis might result in EMT-induced interstitial fibrosis in vivo, because renal fibrosis is inhibited when blocking IL-1 activity using an IL-1R antagonist (Chen et al. 1997). Recently, a positive correlation between NF-κB and activated EMT-related transcription factors, such as TWIST1 and Slug, was described in several human cancers (Liu et al. 2017; Pires et al. 2017). EMT is associated with the loss of epithelial traits and gain of mesenchymal characteristics, including altered cell migration and invasion. In the present study, evaluation of EMT following *TIR8* knockdown in HKC cells and after IL-1β treatment revealed acquisition of a myofibroblast-like phenotype, significantly increased migration and invasion, loss of the epithelial marker E-cadherin, and gain of the mesenchymal marker vimentin. Additionally, IL-1β altered patterns of *TWIST1* and *Slug* expression in HKC/shTIR8 cells due to activation of NF-κB as a transcriptional regulator of EMT-inducing factors related to metastatic progression (Fan et al. 2001). Moreover, as a canonical inducer promoting *TWIST1* and *Slug* expression required for EMT, TGF-β1 significantly increased autocrine signaling in HKC/shTIR8 cells treated with IL-1β. These results supported a protective role for TIR8 in IL-1β-induced EMT in HKC cells.

In agreement with *in vitro* findings, in vivo experiments showed that IL-1β-activated IL-1R/NF-κB signaling might contribute to upregulating TGF-β1 levels and the development of kidney fibrosis. We observed attenuated *TIR8* expression in renal tubular epithelial cells from animal models of UUO-induced renal fibrosis due to high levels of IL-1β and activated NF-κB (p-p65). This suggested that IL-1β might be capable of inducing EMT in renal tubular epithelial cells upon loss of TIR8-specific functions. Furthermore, renal tubular epithelial cells exposed to IL-1β acquired new migration and invasion abilities in vivo that might correlate with movement across the tubular basement membrane and ultimately into the interstitial compartments of the kidney.

**Fig. 7:**
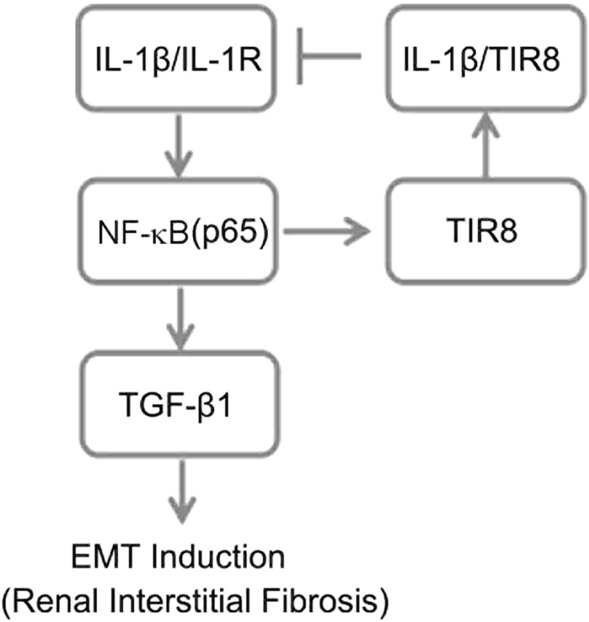
Schematic illustration of TIR8-mediated regulation of IL-1β-induced EMT via IL-1R/NF-κB signaling and negative feedback. Renal tubular epithelial cells maintain a balance between *TIR8* expression and activation of IL-1R signaling under physiological conditions. IL-1β activates NF-κB mediated by IL-1R signaling to increase *TIR8* expression. Overexpressed *TIR8* attenuates IL-1R signaling, and activated NF-κB promotes upregulated TGF-β1 levels, which induces EMT in renal tubular epithelial cells

In summary, we investigated *TIR8* expression in renal tubular epithelial cells following exposure to IL-1β, as well as its regulatory mechanism associated with EMT in renal tubular epithelial cells. We found that *TIR8* was continuously expressed in renal tubular epithelial cells at the physiological levels and upregulated by activation of an NF-κB-mediated pathway during long-term IL-1β stimulus. Additionally, we observed that NF-κB activation and subsequent upregulation of TGF-β1 levels induced EMT in renal tubular epithelial cells. Furthermore, our findings suggested that *TIR8* overexpression might attenuate IL-1R/NF-κB signaling as part of a negative feedback loop (Fig. 7). These results provide new insights into TIR8-mediated negative regulation of IL-1R/NF-κB signaling, which is involved in EMT during RIF development.

## Supplementary Information

Below is the link to the electronic supplementary material.

Supplementary file1 (DOCX 19 KB)
