# Supplementary material for: A negative feedback loop involving NF-κB/TIR8 regulates IL-1β-induced epithelial- myofibroblast transdifferentiation in human tubular cells
Source: J Cell Commun Signal. 2021 May 4;15(3):393–403. doi: 10.1007/s12079-021-00620-8 (PMC8222463; doi:10.1007/s12079-021-00620-8)
Supplement: Supplementary file 1 — Supplementary file1 (DOCX 19 KB) [file 12079_2021_620_MOESM1_ESM.docx]

**Supplementary materials**

**Supplementary Table 1.**

**Oligoes used to knockdown genes.**

| Oligo | Sequences (5'-3') |
| --- | --- |
| shp65 | CACCATCAACTATGATGAGTT |
| ShTIR8-1# | CCATCCAGAACATCAGCTTCT |
| ShTIR8-2# | TGACCAGCACTGAAGTCTATG |
| ShTIR8-3# | GTTCGTGAACTTCATCCTAAA |
| shSc | AATCGCATAGCGTATGCCG |

**Supplementary Table 2.**

**Quantitative RT-PCR primers sequences.**

| Gene | Primers sequences(5′ to 3′) |
| --- | --- |
| TIR8-forward  TIR8-reverse  Slug-forward  Slug-reverse  Twist1-forward  Twist1-reverse  β-actin-forward  β-actin-reverse | CTCCCCGTCTGAAGACCAG  CCCCAATTCCCAATGGAAGC  CCAAGCTTTCAGACCCCCA  TGCAGCTGCTTATGTTTGG  ATTCAAAGAAACAGGGCGTGG  GCAGAGGTGTGAGGATGGTG  GTGGACATCCGCAAAGAC  AAAGGGTGTAACGCAACTAA |
